# RNA-Binding Profiles of CKAP4 as an RNA-Binding Protein in Myocardial Tissues

**DOI:** 10.3389/fcvm.2021.773573

**Published:** 2021-12-23

**Authors:** Hong Zhu, Yanfeng Zhang, Chengliang Zhang, Zhongshang Xie

**Affiliations:** ^1^Department of Cardiovascular Surgery, Xiangya Hospital, Central South University, Changsha, China; ^2^National Clinical Research Center for Geriatric Disorders, Xiangya Hospital, Central South University, Changsha, China

**Keywords:** cardiac fibrosis, cardiac remodeling, iRIP-seq, CKAP4, lncRNA

## Abstract

**Background:** Pathological tissue remodeling such as fibrosis is developed in various cardiac diseases. As one of cardiac activated-myofibroblast protein markers, CKAP4 may be involved in this process and the mechanisms have not been explored.

**Methods:** We assumed that CKAP4 held a role in the regulation of cardiac fibrotic remodeling as an RNA-binding protein. Using improved RNA immunoprecipitation and sequencing (iRIP-seq), we sought to analyze the RNAs bound by CKAP4 in normal atrial muscle (IP1 group) and remodeling fibrotic atrial muscle (IP2 group) from patients with cardiac valvular disease. Quantitative PCR and Western blotting were applied to identify CKAP4 mRNA and protein expression levels in human right atrium samples.

**Results:** iRIP-seq was successfully performed, CKAP4-bound RNAs were characterized. By statistically analyzing the distribution of binding peaks in various regions on the reference human genome, we found that the reads of IP samples were mainly distributed in the intergenic and intron regions implying that CKAP4 is more inclined to combine non-coding RNAs. There were 913 overlapping binding peaks between the IP1 and IP2 groups. The top five binding motifs were obtained by HOMER, in which GGGAU was the binding sequence that appeared simultaneously in both IP groups. Binding peak-related gene cluster enrichment analysis demonstrated these genes were mainly involved in biological processes such as signal transduction, protein phosphorylation, axonal guidance, and cell connection. The signal pathways ranking most varied in the IP2 group compared to the IP1 group were relating to mitotic cell cycle, protein ubiquitination and nerve growth factor receptors. More impressively, peak analysis revealed the lncRNA-binding features of CKAP4 in both IP groups. Furthermore, qPCR verified CKAP4 differentially bound lncRNAs including LINC00504, FLJ22447, RP11-326N17.2, and HELLPAR in remodeling myocardial tissues when compared with normal myocardial tissues. Finally, the expression of CKAP4 is down-regulated in human remodeling fibrotic atrium.

**Conclusions:** We reveal certain RNA-binding features of CKAP4 suggesting a relevant role as an unconventional RNA-binding protein in cardiac remodeling process. Deeper structural and functional analysis will be helpful to enrich the regulatory network of cardiac remodeling and to identify potential therapeutic targets.

## Introduction

Maintenance of normal cardiac function requires fine-tune regulation of myocardial homeostasis that enables the heart to self-adjust under physiological stress and volume load to produce physiological hypertrophic responses and compensatory growth, thus playing a key role in the cardiac development and stress response (1–3). Acquisition of cardiac homeostasis needs normal specific cellular-molecular mechanisms and coordinated intercellular interactions which could be interfered by persistent and harmful external environmental stimuli such as overload, myocardial ischemia, leading to pathological tissue remodeling deviating from homeostasis and causing various cardiac diseases such as ischemic cardiomyopathy, hypertrophic cardiomyopathy, atrial fibrillation and other arrhythmias (4–7). Heart failure caused by these diseases further promotes abnormal myocardial remodeling, which in turn exacerbates heart failure to form a vicious circle (8). Excessive fibrosis, degeneration and loss of cardiomyocytes have been involved in this pathological process (9–11). Although the potentially complex molecular regulatory networks have been extensively studied, our knowledge of pathological cardiac remodeling is still limited (12–14).

Classical RNA-binding proteins (RBPs) have been discovered to participate the regulation of genetic posttranscriptional expression and been essential regulatory molecules to maintain normal cell function and RNA stability (15, 16). These proteins are also implicated in the development of multiple cardiovascular diseases (17, 18). Recently, by high-throughput translation group sequencing, many RBPs associated with myocardial fibrosis have been excavated (19). They have been validated to target transcriptional sequences, control protein expression levels, and to promote cardiac fibroblasts activation into myofibroblasts, which is an important mechanism dominating the progression of dilated cardiomyopathy and heart failure (18, 19). Furthermore, recent efforts have identified a series of non-classical RBPs, expanding our understanding of this category proteins (20, 21). Without classical RNA-binding domains, these RBPs not only bind mRNA, but also interact with non-coding RNA, mediating important biological process such as stress response and energy metabolism (20, 21).

Previous studies have asserted that cytoskeleton-associated protein 4 (CKAP4, CLIMP-63) is a multifunctional trans-membrane protein as a receptor for several ligands (APF, SPA, tPA, DKK-1) (22–25). As an initially discovered functional protein on the endoplasmic reticulum, CKAP4 acts to govern the morphology and quantity of endoplasmic reticulum membrane and mediate the cross-linking of endoplasmic reticulum and microtubules (26, 27). Interestingly, CKAP4 can also form complexes with protein Dicer and be involved in the processing of microRNA precursors (28). In addition, CKAP4 have potential interaction with DNA sequences. For example, CKAP4 enhances the expression of connective tissue growth factor (CTGF/CCN2) gene through binding to its promoter (29). Recent research has demonstrated that CKAP4 as one of cardiac activated-myofibroblast protein markers may be involved in cardiac fibrosis (30).

Here, we assumed that CKAP4 held a role in the regulation of cardiac function and remodeling as an RNA-binding protein. Our study has confirmed decreased expression of CKAP4 in the enlarged atrial myocardium with pathological remodeling. By improved RNA immunoprecipitation and sequencing technology (iRIP-seq), we have revealed certain RNA-binding features of CKAP4 suggesting a relevant role as an unconventional RNA-binding protein in cardiac remodeling process.

## Materials and Methods

### Tissue Preparation

Atrial tissues were obtained from patients with cardiac valvular disease during valvular operations and were rapidly frozen in liquid nitrogen and stored at −80°C for protein extraction and RNA isolation. Atrial tissues of normal size from patients without atrial fibrillation were classified as normal group, and those of enlarged size from patients with atrial fibrillation were classified as remodeling group. The study was approved by the Ethics Committee of Xiangya Hospital, Central South University.

### Immunoprecipitation

Myocardial tissues were irradiated once for 400 mJ/cm^2^, grinded in liquid nitrogen and lysed in ice-cold lysis buffer (1×PBS, 0.5% sodium deoxycholate, 0.1% SDS, 0.5% NP40) with RNase inhibitor (2313,Takara bio, Shiga, Japan) and a protease inhibitor (329-98-6, Solarbio, Beijing, China) on ice for 5 min. RQ I DNase (M610A, Promega, Madison, WI, USA) was added in lysis solution and incubated at 37 °C for 15 min followed by MNase (EN0181, Thermo Fisher Scientific, Waltham, MA, USA) added and incubated at 37°C for 10 min. The mixture was then vibrated vigorously and centrifuged at 13,000 x g at 4°C for 20 min to remove cell debris. The supernatant was incubated with DynaBeads protein A/G (26162, Thermo Fisher Scientific) conjugated with anti-CKAP4 antibody (16686-1-AP, Proteintech, Chicago, IL, USA) or normal IgG at 4°C overnight. Then the beads were washed with Low-salt Wash buffer, High-salt Wash buffer and 1X PNK Buffer, respectively. Following the beads were resuspended in Elution Buffer (50 mM Tris-Cl (PH = 8.0), 10 mM EDTA (PH = 8.0), 1%SDS) and divided into two parts, 4/5 for RNA isolation from CKAP4-RNA complexes and 1/5 for the western blotting assay for CKAP4.

### Western Blot

The sample from immunoprecipitation was resuspended with 40 ul Elution Buffer, incubated at 70°C for 20 min at 1,400 rpm, and then centrifuged at 13,200 x g for a short time. The supernatant was transferred to a new EP tube on a magnetic separator. Next, complexes were eluted in boiling water for 10 min with 1X SDS sample buffer and separated on 10% SDS-PAGE. With TBST buffer (20 mM Tris-buffered saline and 0.1% Tween-20) containing 5% non-fat milk powder for 1 h at room temperature, the Membrane was incubated with primary antibody: CKAP4 antibody (1:1,000, 16686-1-AP, Proteintech), GAPDH antibody (1:2000, CUSABIO, Wuhan, China) and then with HRP-conjugated secondary antibody. Bound secondary antibody (anti-mouse or anti-rabbit 1:10,000) (Abcam, Cambridge, MA, USA) was detected using the enhanced chemiluminescence (ECL) reagent (170506, Bio-Rad, Hercules, CA, USA).

### RNA Extraction and RT-qPCR

The CKAP4-bound RNAs were isolated from the immunoprecipitation sample using TRIzol (15596, Invitrogen, Carlsbad, CA, USA). cDNA libraries were prepared with One-Step gDNA Removal and cDNA Synthesis Mix (AT311-03, Transgen Biotech, Beijing, China). qPCR was prepared with HieffTM qPCR SYBR^®^ Green Master Mix (11201, YEASEN, Shanghai, China) and detected on Q6 real-time PCR detection system. The information of primers used for RT-qPCR is presented in Supplemental Material.

For assessing the expression of CKAP4 in human myocardial tissues, we used housekeeping gene GAPDH (glyceraldehyde-3-phosphate dehydrogenase) as a control gene. cDNA synthesis was conducted by standard procedures for following real-time quantification PCR, which was performed with the HieffTM qPCR SYBR Green Master Mix (Low Rox Plus) (11202, YEASEN, Shanghai, China). The results were processed using 2-ΔΔCT method.

### iRIP-Seq Library Preparation and Sequencing

The CKAP4-bound RNAs from the immunoprecipitation sample were isolated using TRIzol (Invitrogen). cDNA libraries were prepared with the KAPA RNA Hyper Prep Kit (KK8541, KAPA Biosystems, Boston, USA) according the manufacturer's procedure, followed by high-throughput sequencing on an Illumina Xten platform for 150 bp paired-end sequencing.

### Data Analysis

Paired-end sequencing reads were aligned onto the human genome, only uniquely mapped reads were used for the following analysis. “ABLIRC” strategy was applied to identify the binding regions of CKAP4 on genome (31). Reads with at least 1 bp overlap were clustered as peaks. For each gene, computational simulation was performed to randomly generate reads with the same number and lengths as reads in peaks. The outputting reads were further mapped to the same gene to generate random max peak height from overlapping reads. The whole process was repeated for 500 times. All the observed peaks with heights higher than those of random max peaks (*p* < 0.05) were selected. The IP and input samples were analyzed by the simulation independently, and the IP peaks that have overlap with input peaks were removed. The target genes of IP were finally determined by the peaks and the binding motifs of CKAP4 were called by HOMER software.

### Functional Enrichment Analysis

To sort out functional categories of peak-associated genes (target genes), Gene Ontology (GO) terms and KEGG pathways were identified using KOBAS 2.0 server (32). Hypergeometric test and Benjamini-Hochberg FDR controlling procedure were applied to define the enrichment of each term.

### Availability of Data and Materials

The raw data are available under GEO Series accession number GSE161695.

## Results

### iRIP-Seq of CKAP4 in Myocardial Tissues

**Figure 1:**
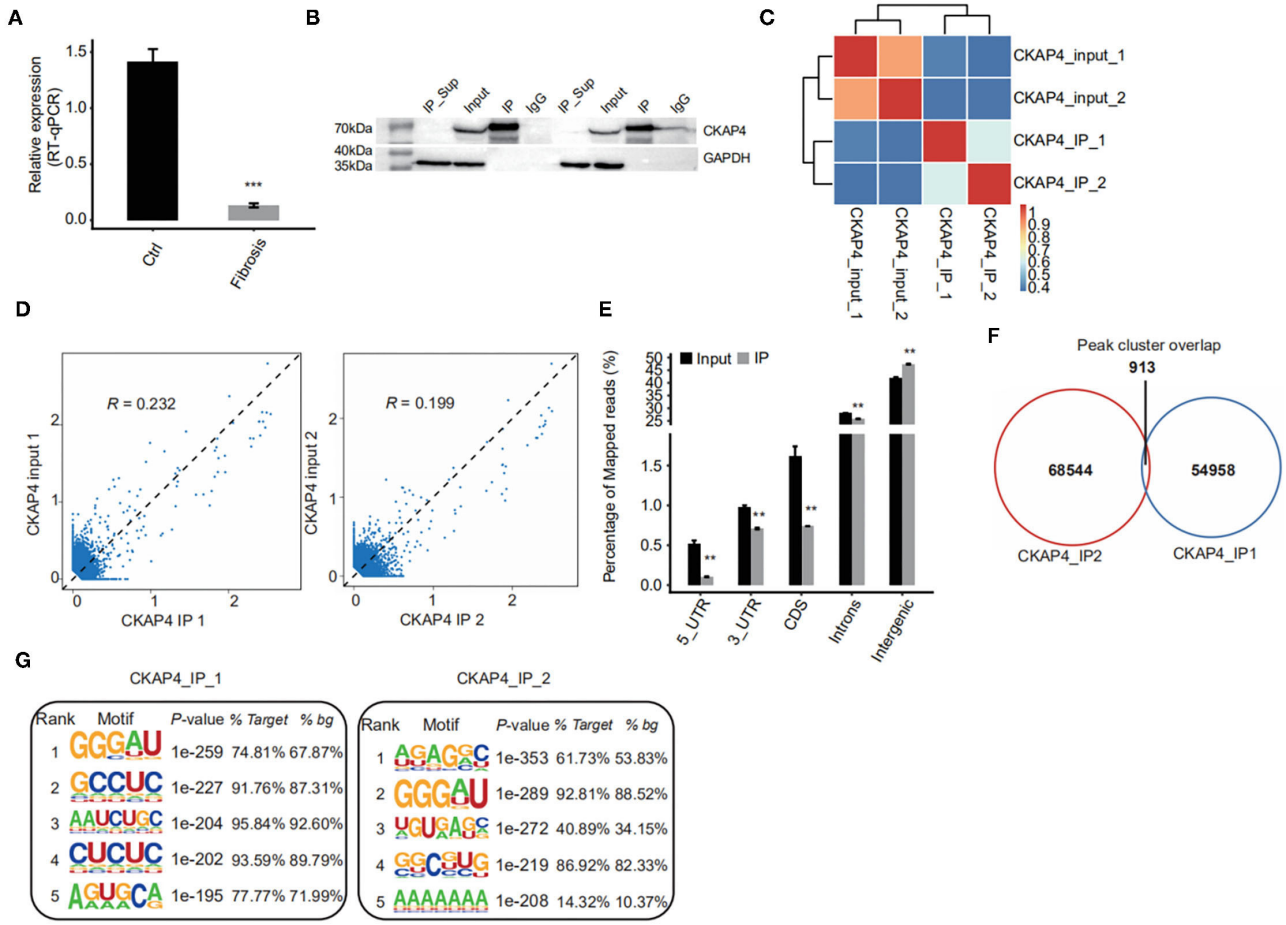
iRIP-seq of CKAP4 in myocardial tissues. **(A)** CAP4 RNA level was quantified by qRT-PCR. Error bars represent mean ± SEM. ***p < 0.001. **(B)** CKAP4 protein detection by western blot. **(C)** Heat map shows the hierarchically clustered Pearson correlation matrix resulted from comparing the transcript expression values for input and CKAP4 IP samples. **(D)** Scatter plot of reads abundance across reference genome in paired samples. **(E)** Reads distribution across reference genome. Error bars represent mean ± SEM. **p < 0.01. **(F)** Venn diagram of peaks identified from the IP1 and IP2 samples. **(G)** Top 5 overrepresented CKAP4-binding motifs identified by Homer from the iRIP-seq data.

Using improved RNA immunoprecipitation and sequencing (iRIP-seq, improved RIP-seq), we sought to analyze the RNAs bound by CKAP4 in normal atrial muscle (IP1 group) and remodeling fibrotic atrial muscle (IP2 group) from patients with cardiac valvular disease. Quantitative PCR proved that CKAP4 was differentially expressed in two groups, and the expression was significantly down-regulated in the remodeling fibrosis group (Figure 1A). Western blotting further confirmed the difference of CKAP4 protein expression level between the two groups, and also showed the binding specificity of CKAP4 antibody and the effectiveness of immunoprecipitation technology (Figure 1B). Correlation analysis and hierarchical clustering analysis between samples suggested that the IP group and the input group had significant differences in the CKAP4-bound RNA and their respective characteristics (Figure 1C). By statistically analyzing the distribution of binding peaks in various regions on the reference human genome, we found that the reads of IP samples on the reference genome were mainly distributed in the intergenic and intron regions, and were enriched relative to the input group, which implies that CKAP4 is more inclined to combine non-coding RNAs (Figures 1D,E). The Venn diagram showed that there are 913 overlapping binding peaks between the IP1 and IP2 groups (Figure 1F). We used HOMER (Hypergeometric Optimization of Motif EnRichment) to perform motif analysis on the binding peaks obtained by the ABLIRC analysis method (31) to obtain the top five binding motifs in the IP samples, where GGGAU was the binding sequence that appeared simultaneously in both groups, suggesting that this sequence may be CKAP4 Preferred binding sequences (Figure 1G).

### Binding Peak-Related Gene Cluster Enrichment Analysis

**Figure 2:**
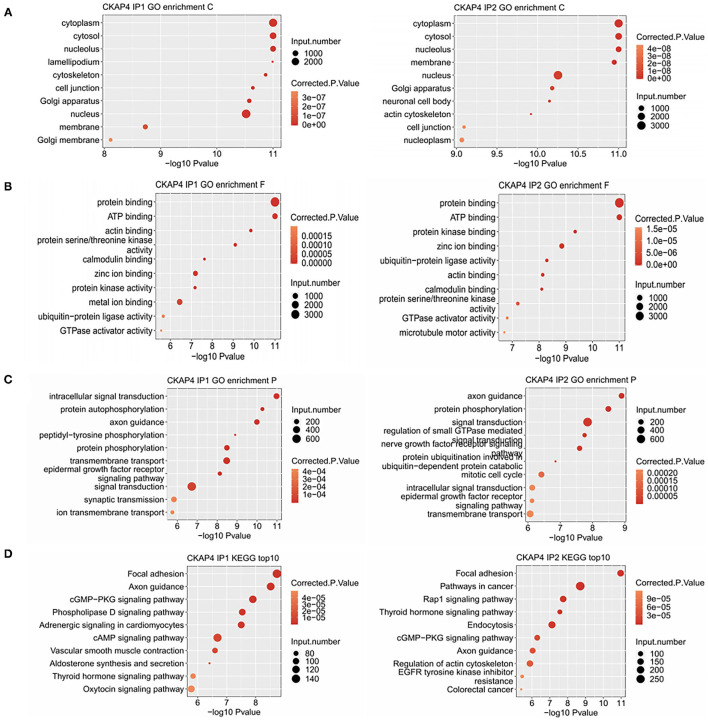
Binding peak-related gene cluster enrichment analysis. Bubble plot showed the top 10 enriched GO cellular component **(A)**, molecular function **(B)**, biological process **(C)** and KEGG **(D)** categories for the CKAP4-bound genes.

Using Gene Ontology, we demonstrated the top 10 GO terms with their corrected p-values (Figures 2A–C). Cellular component categories showed that CKAP4-bound genes were mainly existed in cytoplasm, nucleus, cytoskeleton and cell membrane (Figure 2A). These genes were mainly involved in biological processes such as signal transduction, protein phosphorylation, axonal guidance, and cell connection (Figure 2C). The signal pathways ranking most varied in the remodeling myocardium group (IP2) compared to the normal atrial muscle group (IP1) were relating to mitotic cell cycle, protein ubiquitination and nerve growth factor receptors (Figure 2C). The results of KEGG pathway enrichment analysis were illustrated in Figure 2D in which focal adhesion, axonal guidance, cGMP-PKG signaling pathway were common in both groups.

### Characteristics of CKAP4 Binding to Non-coding RNAs

**Figure 3:**
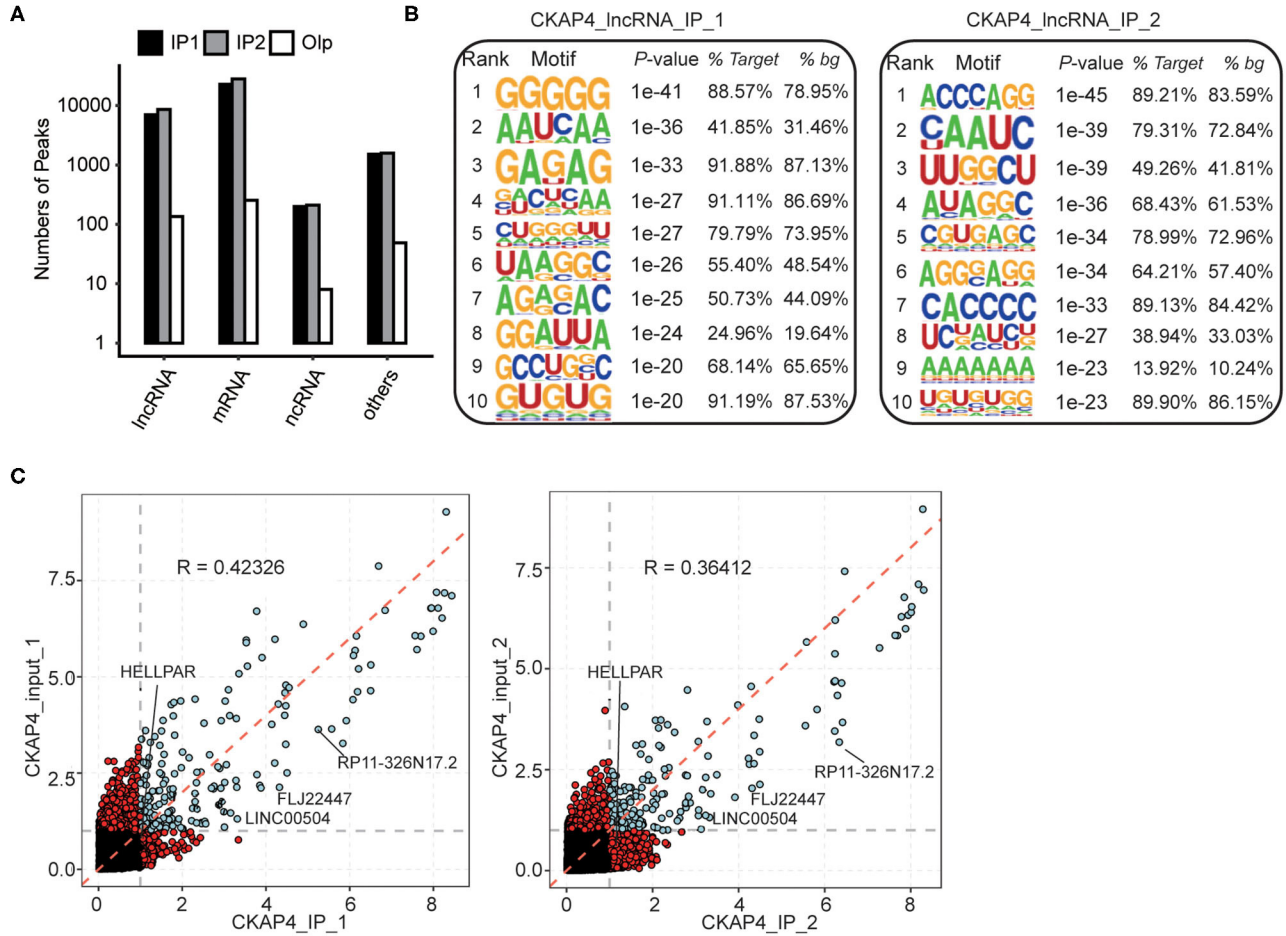
Peak analysis revealed the lncRNA-binding features of CKAP4. **(A)** Bar plot showed the peak numbers of different RNA types. **(B)** Motif analysis showed the top 10 preferred bound motifs of lncRNAs by HOMER software. **(C)** Scatter plot showed the gene RPKM (Reads per kilo base of a gene per million reads) in paired samples. Red points represented the specifically expressed genes in IP or input samples.

In our results, more fascinating is the ability of CKAP4 interacting with non-coding RNAs, especially the long non-coding RNAs (lncRNAs) (Figure 3A). HOMER motif analysis revealed the top 10 binding-motifs on lncRNAs tended to enrich base G, A, C (Figure 3B). Through gene annotation and correlation analysis of binding-peaks reads, we found that at least four lncRNAs including LINC00504, FLJ22447, RP11-326N17.2 and HELLPAR were CKAP4 potential binding lncRNAs in myocardial tissues (Figure 3C).

### CKAP4 Preferentially Binds to lncRNAs in Remodeling Myocardial Tissues

**Figure 4:**
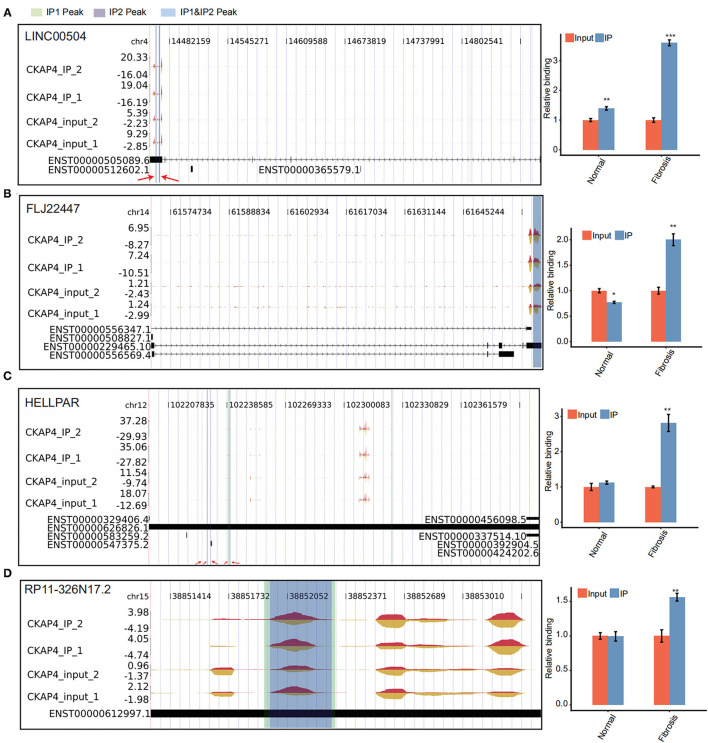
CKAP4 preferentially binds to lncRNAs in remodeling myocardial tissues. **(A–D)** IGV-sashimi plot showed CKAP4 binding sites across lncRNAs, including LINC00504 **(A)**, FLJ22447 **(B)**, HELLPAR **(C)** and RP11-326N17.2 **(D)**. RIP-qPCR validated the binding reliability of CKAP4 with these lncRNAs (right). The asterisk (*) indicates *p < 0.05, **p < 0.01, ***p < 0.001.

We further delved into the binding sites of CKAP4 with four lncRNAs including LINC00504, FLJ22447, RP11-326N17.2 and HELLPAR. The localization of these sites on genome is detailed in Figures 4A–D. Some sites overlap in the normal and remodeling myocardial groups and are considered to be more credible binding sites. Finally, we used quantitative PCR to verify the binding reliability of CKAP4 with these lncRNAs. The results suggest that CKAP4 differentially bind lncRNAs in remodeling myocardial tissues when compared with the control group (Figures 4A–D, right), indicating that the interaction between CKAP4 and lncRNAs may be related to myocardial remodeling such as fibrosis.

### CKAP4 Expression Is Down-Regulated in Remodeling Fibrotic Myocardium

**Figure 5:**
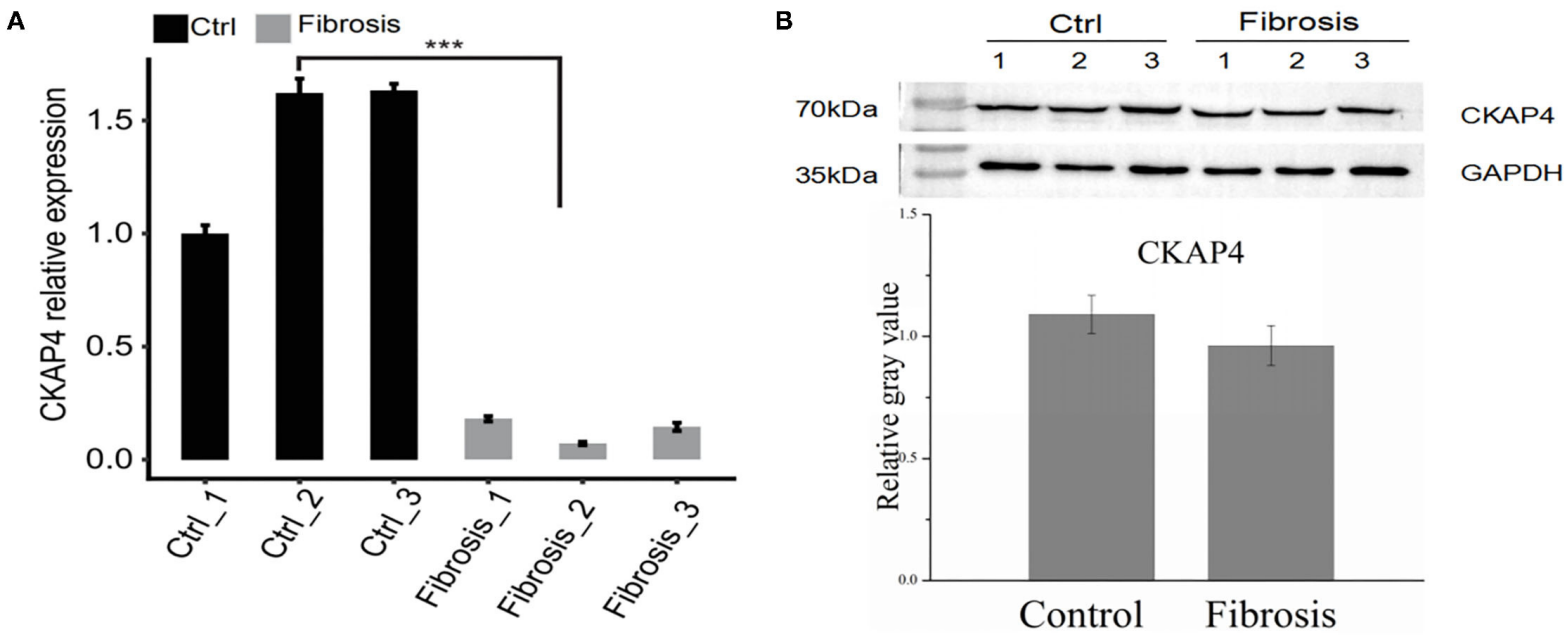
CKAP4 expression is down-regulated in remodeling fibrotic myocardium. Quantitative PCR **(A)** and Western blotting **(B)** results indicated the mRNA and protein expression of CKAP4 in remodeling fibrotic myocardial samples (*n* = 3) decreased significantly relative to control samples (*n* = 3). ***p < 0.001.

we selected right atrial specimens from patients with heart valve disease for quantitative PCR detection (Figure 5A) and Western blotting (Figure 5B) to identify CKAP4 mRNA and protein expression levels. We found that the mRNA and protein expression of CKAP4 in remodeling myocardial samples from significantly enlarged right atrium decreased relative to normal-size right atrial specimens.

## Discussion

Although previous studies have shown that CKAP4 is a multifunctional protein (33), few reports of its ability to bind to RNAs were published. A recent study demonstrates that lncRNA SENCER in vascular cells can bind to CKAP4 non-classical RBD (RNA binding domain) to influence its interaction with membrane protein CDH5(VE-cadherin), thereby stabilizing the latter and maintaining the integrity of endothelial cell adhesion junctions (34). Knockout SENCER enhances protein-protein interactions between CKAP4 and CDH5 which facilitates CDH5 internalization, therefore impairs the adhesion function of endothelial cells (34). Interestingly, in remodeling cardiac tissues, we have found four binding peaks of CKAP4 with CDH5 transcripts (data not shown), but not in normal myocardial tissues. This suggests that during cardiac remodeling, CKAP4 may affect adhesion function and permeability of endothelial cells by regulating location and function of CDH5. In addition, our research reveals that CKAP4 has a broader unknown RNA-binding potential. We believe its interactions with mRNAs and lncRNAs can not only regulate the RNA network, but also in turn may affect its own expression level, cell localization and complex functions.

Using single-cell sequencing techniques, Monika M.Gladka et al. have confirmed that CKAP4 is a protein marker for activated myofibroblasts in ischemic injury myocardium (30). Inhibition of CKAP4 expression increases the expression of TGF-β-stimulated fibroblast activation-related genes implying that CKAP4 functions to weaken the expression of these genes (30). However, another study has reported that CKAP4 Promotes activation of atrial fibroblasts (35). The seemingly contradictory outcomes of these two studies are both originated from *in vitro* cell model. Our results indicate that CKAP4 expression is downregulated in the enlarged myocardium caused by human valvular diseases, which may be associated with myocardial fibrosis. We speculate that the expression level of CKAP4 in tissue is different from that at cellular level. Also CKAP4 may present different expression levels in tissues at different stages of the development of fibroblast transformation into myofibroblasts in which CKAP4 may be up-regulated in the very early stage of fibrosis and down-regulated in the later stage. In any case its specific role in the process of myocardial fibrosis needs further study.

Based on cluster analysis of CKAP4 binding peak-related genes, the localization of these genes is mainly in the cytoplasm, cytoskeleton, membrane bodies, and also in the nucleus. This is consistent with the function of CKAP4 as a transmembrane and framework-related protein. As one of the most abundant proteins in the flat cystic membrane around the endoplasmic reticulum, CKAP4 has higher expression levels in highly active secretory cells (27). It often interacts with other proteins and ribosomes to form polyribosomes on coarse endoplasmic reticulum membranes (27). All these studies indicate that CKAP4 is involved in the synthesis, transport and secretion of secretory proteins and membrane proteins. Furthermore, as one of the endoplasmic reticulum mRNA-related proteins and mRNA receptors, CKAP4 mediates mRNA to be anchored to the endoplasmic reticulum (36). The potential mechanism so far is not clear in detail, although supposed to be independent of ribosomes and translation process (36). We speculate that CKAP4 may directly bind to the mRNA of some secreted proteins and membrane proteins to localize them on the endoplasmic reticulum.

According to our results, the most important function of CKAP4 targeted genes is the protein-binding function, which is the most common role of secretory and membrane proteins. Through further functional and pathway analysis of binding targets, we have found that CKAP4 is associated with signal transduction, protein phosphorylation, axonal orientation, and cellular junction. Axon orientation involves the regulation of skeletal proteins such as actin and microtubules (37, 38). As one of the cell junctions, Focal adhesion is closely related to integrins (39). As an RNA-binding protein, How CKAP4 could participate in the regulatory network of cytoskeleton proteins and integrins, and how it affects myocardial remodeling requires more in-depth investigation.

Among targeted coding genes, zinc finger protein ZNF771 is most pronounced one with the most number of reads in the CKAP4-specific binding peaks in the remodeling myocardium group (IP2) (data not shown), and the function of this gene is unknown until now. Based on enrichment analysis of mitotic cycle-related genes of CKAP4 targets, MNAT1, ALMS1, HDAC1 have been dug out (data not shown). These genes have been found to be participated in the events of heart failure, cardiomyopathy and cardiac fibrosis in previous researches (40–45).

Our primary exploration suggests that CKAP4 is a non-classical RNA-binding protein. In addition to mRNA, lncRNAs are also its preferred binding targets. Our results confirm lncRNA FLJ22447 is an enriched binding target of CKAP4 in remodeling myocardium. There is evidence that lncRNA FLJ22447 expression is upregulated in tumor-associated stromal fibroblasts of oral squamous cell carcinoma, stabilizing IL-33 by inhibiting the autophagic lysosomal pathway, thereby promoting tumor cell proliferation (46). IL-33 is considered as a myocardial protective factor, which can inhibit cardiomyocyte apoptosis, reduce myocardial fibrosis and inflammatory response, and improve survival and function of ischemic myocardium (47, 48). Whether interaction between CKAP4 and lncRNA FLJ22447 could regulate myocardial remodeling by governing IL-33 expression is worth exploring, which is our next research program. Some other lncRNAs including HELLPAR, RP11-326N17.2 and LINC00504 are potential preferred binding targets of CKAP4, while their function is currently unknown.

Our study was limited to intrinsic defects in iRIP-RNA sequencing techniques and small quantities of tissue samples. Lack of cellular and animal experiments limited current research mainly to bio-information analysis. More samples and multi-level investigation are beneficial to obtain more reliable and concrete results.

## Conclusion

In brief, CKAP4 is an impressive multifaceted protein. Previous studies have unraveled the protein-protein or protein-DNA interactions that CKAP4 is involved in, our study reveals that CKAP4 may be involved in a variety of biological processes through non-classical protein-RNA interactions in cardiac tissues. Deeper structural and functional analysis will be helpful to enrich the regulatory network of cardiac remodeling and to identify potential therapeutic targets.

## Data Availability Statement

The datasets presented in this study can be found in online repositories. The names of the repository/repositories and accession number(s) can be found in the article/Supplementary Material.

## Ethics Statement

The studies involving human participants were reviewed and approved by the Ethics Committee of Xiangya Hospital, Central South University. The patients/participants provided their written informed consent to participate in this study. Written informed consent was obtained from the individual(s) for the publication of any potentially identifiable images or data included in this article.

## Author Contributions

HZ: collection of samples, experiments, data analysis, manuscript writing, and final approval of manuscript. YZ and CZ: experiments. ZX: concept and design, data analysis and interpretation, manuscript writing, and final approval of manuscript. All authors contributed to the article and approved the submitted version.

## Conflict of Interest

The authors declare that the research was conducted in the absence of any commercial or financial relationships that could be construed as a potential conflict of interest.

## Publisher's Note

All claims expressed in this article are solely those of the authors and do not necessarily represent those of their affiliated organizations, or those of the publisher, the editors and the reviewers. Any product that may be evaluated in this article, or claim that may be made by its manufacturer, is not guaranteed or endorsed by the publisher.

## Abbreviations

CKAP4: Cytoskeleton-associated protein 4
CLIMP-63: The microtubule-binding 63-kDa cytoskeleton-linking membrane protein
CTGF/CCN2: Connective tissue growth factor
iRIP-seq: Improved RNA immunoprecipitation and sequencing
lncRNAs: Long non-coding RNAs
RBPs: RNA-binding proteins
MNAT1: CDK-activating kinase assembly factor MAT1
ALMS1: Alstrom syndrome protein 1
HDAC1: Histone deacetylase 1
APF: Anti-proliferative factor
SPA: Surfactant protein A
tPA: Tissue plasminogen activator
DKK-1: Dickkopf-1
TGF-β: Transforming growth factor-β
CDH5: Cadherin 5.
