# Supplementary material for: RNA-Binding Profiles of CKAP4 as an RNA-Binding Protein in Myocardial Tissues
Source: Front Cardiovasc Med. 2021 Dec 23;8:773573. doi: 10.3389/fcvm.2021.773573 (PMC8733325; doi:10.3389/fcvm.2021.773573)
Supplement: Supplementary file 1 [file Table_1.DOCX]

| CKAP4 | F 5' CAGCCGGATCAGCGAAGT 3'  R 5' TGTGAAGATGGCGATGTTGT 3' |
| --- | --- |
| LINC00504 | F 5' GGCTTTGGTATCAGGATG 3'  R 5' AGAGGTACATGGAGGAGC 3' |
| HELLPAR | F 5' AAACTATACTACAAGGCTACG 3'  R 5' TGGTGTTATTTCTGAGGC 3' |
| FLJ22447 | F 5' ACCCAAATGTCCATCAGT 3'  R 5' GCTTCATCCATGTCCCTA 3' |
| RP11-326N17.2 | F 5' GAATAGTGCTGCAATAAAC 3'  R 5' AGAAATACCATTTGACCC 3' |

Table S1 Primers used for RT-qPCR
